# Learning and memory with neuropathic pain: impact of old age and progranulin deficiency

**DOI:** 10.3389/fnbeh.2013.00174

**Published:** 2013-11-22

**Authors:** Boris Albuquerque, Annett Häussler, Elisabetta Vannoni, David P. Wolfer, Irmgard Tegeder

**Affiliations:** ^1^Department of Clinical Pharmacology, pharmazentrum frankfurt, Goethe-University Hospital Frankfurt am MainFrankfurt am Main, Germany; ^2^Faculty of Medicine, Institue of Anatomy, University of ZurichZurich, Switzerland; ^3^Department of Health Sciences and Technology (D-HEST), Institute of Human Movement Sciences and Sport, ETH ZurichZurich, Switzerland

**Keywords:** sciatic nerve injury, memory, pain, progranulin, aging

## Abstract

Persistent neuropathic pain is a frequent consequence of peripheral nerve injuries, particularly in the elderly. Using the IntelliCage we studied if sciatic nerve injury obstructed learning and memory in young and aged mice, each in wild type and progranulin deficient mice, which develop premature signs of brain aging. Both young and aged mice developed long-term nerve injury-evoked hyperalgesia and allodynia. In both genotypes, aged mice with neuropathic pain showed high error rates in place avoidance acquisition tasks. However, once learnt, these aged mice with neuropathic pain showed a significantly stronger maintenance of the aversive memory. Nerve injury did not affect place preference behavior in neither genotype, neither in young nor aged mice. However, nerve injury in progranulin deficient mice impaired the learning of spatial sequences of awarded places, particularly in the aged mice. This task required a discrimination of clockwise and anti-clockwise sequences. The chaining failure occurred only in progranulin deficient mice after nerve injury, but not in sham operated or wildtype mice, suggesting that progranulin was particularly important for compensatory adaptations after nerve injury. In contrast, all aged mice with neuropathic pain, irrespective of the genotype, had a long maintenance of aversive memory suggesting a negative alliance and possibly mutual aggravation of chronic neuropathic pain and aversive memory at old age.

## Introduction

Chronic pain seriously reduces the quality of life and impacts on many aspects of daily living. Particularly following nerve injury or neuritis, elderly people have a higher risk to develop chronic neuropathic pain than younger adults, for unknown reasons (Charest and Kenny, 2000; Hochman et al., 2011; Bouhassira et al., 2012) and hence older people may be at a higher risk for the sequelae of chronic pain including emotional and social disability. Chronic pain has been suggested to constitute a kind of learning phenomenon in that the nociceptive experience is engraved into nociceptive signaling pathways by lowering activation thresholds, eliciting spontaneous activity, alteration of synapses and glial activation (Melzack et al., 2001; Ji et al., 2003; Zhao et al., 2006; Zhuo, 2007; Denk and McMahon, 2012). While the concept of “pain-memory” is well-accepted it has not been systematically studied if chronic pain impacts on cognition and memory besides the pain itself (Jongsma et al., 2011) and if chronic pain may constitute a “pro-aging” risk factor. Conversely, there is some evidence that the likelihood for chronic pain may be enhanced in neurodegenerative diseases, particularly Parkinson's disease (Tinazzi et al., 2008; Zambito Marsala et al., 2011; Borsook, 2012), but it is not known whether chronic pain in afflicted patients may accelerate disease progression.

Progranulin is a multi-functional secreted protein with neuroprotective functions and its upregulation after nerve injury likely contributes to those adaptations, which combat the development of chronic pain (Lim et al., 2012). Loss-of-function mutations of progranulin in humans are associated with ubiquitin positive, Tau-negative frontotemporal dementia and some other neurodegenerative diseases (Baker et al., 2006; Cruts et al., 2006; Mackenzie et al., 2006). It has not been studied if these patients experience stronger or longer lasting pain. The exact functions of progranulin in neurons are still unknown. From its interactions with other proteins and the pathology of other forms of frontotemporal dementia (Sleegers et al., 2010; Rademakers et al., 2012) one may hypothesize that is it involved in protein quality control and trafficking (Hu et al., 2010; Almeida et al., 2011). Progranulin deficient mice develop a premature age-dependent gliosis and lipofuscinosis, which is associated with mild deficits of memory functions in old mice (>1.5 years) (Ahmed et al., 2010; Yin et al., 2010a,b). Younger animals appear quite normal, but we found previously that sciatic nerve injury causes stronger motor dysfunctions and nociceptive hypersensitivity in progranulin deficient mice as compared to the controls (Lim et al., 2012) suggesting that they may also be more vulnerable to the pathological sequelae of neuropathic pain and hence may represent a model to study the potentially mutual negative impact of chronic neuropathic pain and memory dysfunctions.

We therefore assessed the behavior in young and aged progranulin deficient and control mice in place avoidance learning and extinction, in place preference learning and in spatial sequencing tasks after a sciatic nerve injury as compared to sham treated mice. To find subtle differences, learning paradigms were tested in home cage environments employing the IntelliCage (Voikar et al., 2010; Endo et al., 2011; Codita et al., 2012). The data shows that nerve injured aged mice have difficulties in avoidance acquisition, but once learnt, retain the aversive memory longer than sham treated or young mice. In addition, the performance in spatial sequencing tasks was compromised in nerve injured progranulin deficient mice, particularly in the aged group. The results suggest that neuropathic pain at old age may negatively affect cognition and memory functions and may impair the extinction of bad memories, which in turn might further strengthen nociceptive hypersensitivity.

## Methods

### Animals

The experiments adhered to the guidelines of the Committee for Research and Ethical Issues of the International Association for the Study of Pain (IASP). They were approved by the local Ethics Committee for Animal Research (Darmstadt, Germany) and adhered to the guidelines of GV-SOLAS for animal welfare in science.

Female C57BL/6J mice (Harlan Winkelmann, Borchen, Germany) and female homozygous progranulin deficient mice (Grn^−/−^) (Yin et al., 2010a) were 9–12 weeks (young) or 10–13 months old (aged) at the time of surgery. Female mice were used to avoid losses due to male hostility, which regularly occurs in groups of aged male mice. Progranulin deficient mice have a pure C57BL/6 genetic background so that age-matched C57BL/6 mice were used as wild type control mice. To avoid alterations due to age-dependent gliosis, which was found in progranulin deficient mice at ages >18 months (Ahmed et al., 2010), we used the middle-aged mice. Mice were housed three to five per cage at constant room temperature (21 ± 1°C) before the experiments, standard diet and a regular light/dark schedule with light on from 7:00 a.m. to 7:00 p.m. Food and water was available *ad libitum* except for the experimental sessions in the IntelliCage requiring a restriction of drinking periods. The controls were housed for 3–4 weeks under identical conditions as the progranulin deficient mice prior to the start of experiments to avoid housing or diet dependent differences. The mean body weight was 20.4 ± 1.5 g and 21.8 ± 1.0 g for young (12 weeks old) female C57BL/6J and Grn^−/−^ mice, respectively, and 26.8 ± 2.5 g and 29.3 ± 2.1 g for aged (12 months) female C57BL/6J and Grn^−/−^mice, respectively. Mice had a spared nerve injury of the sciatic nerve (SNI) or sham surgery, then performed the IntelliCage experiments and were subsequently analyzed for pain sensitivity. The time course of nociception encompassing the period of the IntelliCage experiments was additionally analyzed in a different group of mice because mice must not be disturbed during the IntelliCage experiments.

### Spared nerve injury of the sciatic nerve and transponder implantation

Surgery was carried out under 1.5–2% isoflurane anesthesia. Two of the three peripheral branches of the sciatic nerve, the common peroneal and the tibial nerves, were ligated with silk (6–0) and distally transected, leaving the sural nerve intact (Decosterd and Woolf, 2000). In sham animals, the sciatic nerve was exposed but not injured. Adaptation in the IntelliCage started 14 days after surgery. Radio-frequency identification (FTID) transponders were subcutaneously implanted under isoflurane anesthesia 1 week after SNI surgery.

### Behavioral analysis of nociception and motor functions

Behavioral tests were performed without knowledge of mouse age and genotypes. After habituation to the testing cages, mice were tested for their reaction latencies to mechanical, cold and heat stimulation. A dynamic Plantar Aesthesiometer (Ugo Basile, Italy) was used to assess mechanical nociception. In this test a von Frey-like filament is pushed against the plantar side of the hind paw with linear ascending force (0–5, 0.5 g/s) and is then maintained at 5 g until a strong and immediate withdrawal occurs. The paw withdrawal latency was the mean of three consecutive trials with intervals of at least 30 s. The acetone test was used to measure cold allodynia. After application of a drop of acetone to the plantar hind paw nerve injured mice lick, lift and shake the paw. The time spent with these reactions was monitored with a stop watch for a period of 90 s starting immediately after application of acetone. Heat hyperalgesia was assessed by recording the paw withdrawal latency in the Hargreaves Test (IITC 390 Plantar Test), in which a radiant heat source is placed underneath the hind paw with a mirror system and applies radiant heat upon pressure of a button. The heating is automatically stopped upon paw withdrawal and the latency time is monitored. Three tests with intervals of at least 5 min were performed and results averaged. Motor functions were analyzed by testing RotaRod running behavior (Ugo Basile) at a constant speed. Mice were placed on a rotating bar and the time until they fall off was recorded as the “fall off latency,” with a cut off of 90 s. Four tests were performed and averaged.

### IntelliCage

**Figure 1:**
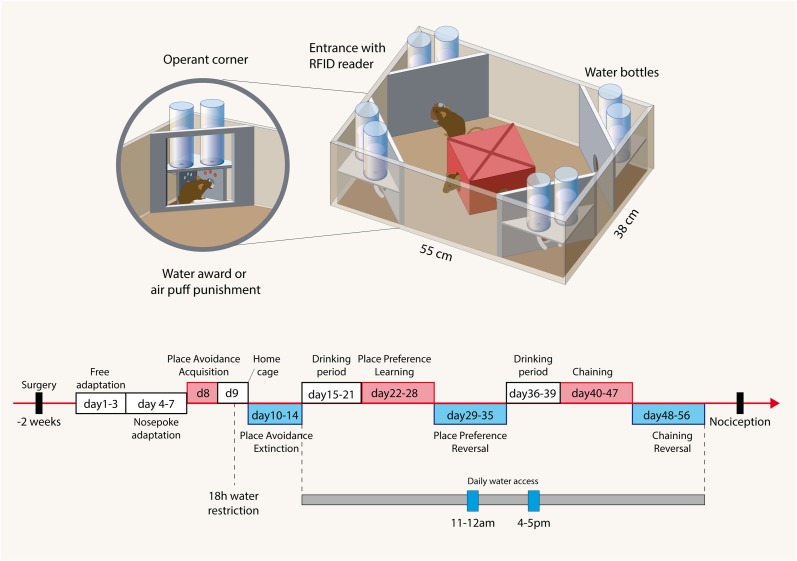
**Illustration of the IntelliCage and the time course of experimental tasks**. Cage: The IntelliCage consists of four operant corners, each with two water bottles, sensors and doors that control the access to the water bottles. Mice are tagged with radio-frequency identification transponders (RFID), which are read at the corner entrance. Inside the corners, there are two holes with water bottles, which can be opened and closed by automated doors. Mice have to make a nosepoke to open the doors for water access. The IntelliCage is controlled by a computer. The number and duration of corner visits, nosepokes, licks, and contact times with the nipples of the water bottles are automatically recorded. Time schedule: The spared nerve injury and sham surgeries were performed 2 weeks before starting the free adaptation in the IntelliCage with free access to water in all corners. During “nosepoke adaptation” mice had to perform a nosepoke to get water, which was possible in every corner. In the “avoidance acquisition” mice received an air-puff punishment and did not get water in one of the corners. During “avoidance extinction” no air-puff punishments were applied and water was available in every corner. In “drinking periods” mice were adapted to get water only at 11–12 a.m. and 4–5 p.m. These restricted drinking times were maintained up to the end of the experiments and provided some synchronization of the behavior. During “place preference learning” mice got water in only one corner. After learning to prefer this corner, the water awarding corner was switched to the respective opposite corner (“place preference reversal”). After cleaning the cage and removing cues mice were then re-adapted to the drinking periods, which were maintained for “chaining” and “reversed chaining” tasks. In these experiments mice got water in one corner either in a clockwise or anti-clockwise sequence of the corners. After 8 days the direction was inverted. Tasks are summarized in Table 1.

The IntelliCage (NewBehavior AG, Zurich, Switzerland) (Krackow et al., 2010; Voikar et al., 2010) consists of four operant corners, each with two water bottles, sensors, light-emitting-diodes (LEDs) and doors that control the access to the water bottles (Figure 1). The system fits into a large cage (20 × 55 × 38 cm, Tecniplast, 2000P). Four triangular red shelters (Tecniplast) are placed in the middle to serve as sleeping quarters and as stands to reach the food. The floor is covered with thick bedding.

**Table 1:** **Behavioral tasks**.

**Task name**	**Task description**	**Punishment**	**Duration**	**Drinking time**
Avoidance acquisition	To avoid one corner where water access was blocked and trials punished	Yes air puff	24 h	No restriction
Avoidance extinction	To remember the previously punished corner without reinforcement, i.e., without punishment and free water in each corner	No	4 days	No restriction
Preference learning	To prefer one corner where a water award was provided	No	7 days	Restricted to 11–12 a.m. and 4–5 p.m.
Preference reversal (i.e., Relearning of opposite corner)	To prefer a novel corner on the side opposite to the previous awarding corner	No	7 days	Restricted to 11–12 a.m. and 4–5 p.m.
Chaining learning	To learn a clockwise (or anticlockwise) sequence of awarding corners	No	8 days	Restricted to 11–12 a.m. and 4–5 p.m.
Chaining reversal (i.e., relearning of opposite sequence direction)	To relearn a novel chaining of awarding corners after switch of the sequence (clockwise to anti-clockwise and vice versa)	No	10 days	Restricted to 11–12 a.m. and 4–5 p.m.

Mice are tagged with RFID-transponders, which can be read with an RFID antenna, which is integrated at the corner entrance. Inside the corners, there are two holes with water bottles, which can be opened and closed by automated doors. Mice have to make a nosepoke to open the doors for water access. The IntelliCage is controlled by a computer with IntelliCage Plus software, which executes pre-programmed experimental schedules. The number and duration of corner visits, nosepokes, licks, and contact times with the nipples of the water bottles are automatically recorded without the need for any handling of the mice during the recording times. Sixteen mice were housed in each cage. Mice were grouped into young and aged mice with and without a sciatic nerve lesion (SNI vs. sham). Each group consisted in 8 mice. One mouse in the aged SNI-treated control and Grn^−/−^ groups had to be removed during the course of the experiments because of RFID dysfunctions.

### Behavioral tasks

The time schedule for the IntelliCage experiments is shown in Figure 1 and followed established protocols (Krackow et al., 2010; Voikar et al., 2010; Endo et al., 2011). Two weeks after surgery mice were adapted to the system for 3 days with free access to every corner, with all doors open and water and food *ad libitum*. This free adaptation was followed by 4-days “nosepoke adaptation,” during which the doors were closed, the first nosepoke of the visit opened the door for 5 s and in order to drink more, the animals had to leave the corner and start a new visit. After this “nosepoke adaptation,” corners were randomly assigned to each 4 animals for the avoidance conditioning. In the assigned corner, the nosepoke triggered an air-puff (~ 0.8 bar, 1-s) until the animal left the corner and the doors in this corner remained closed. The “avoidance acquisition” lasted for 24 h. At completion, mice were returned to their home cages for 1 day with water restriction for the last 18 h prior to the return to their IntelliCage for the analysis of the extinction of the avoidance behavior. The water restriction was used to ensure that the mice were immediately looking for water despite the previous punishments. In the “avoidance extinction” no air-puff punishments were applied and water was available in each corner upon nosepoking. At the end of this test which lasted for 5 days, the IntelliCages were cleaned and environmental cues thereby removed.

The mice were then adapted for 7 days to the “drinking-session” protocol, in which drinking was allowed exclusively between 11 and 12 a.m. and 4 and 5 p.m. Outside of these times the doors remained closed. Subsequently, mice were conditioned for 7 days to one corner, in which they got a water-reward. Drinking during this “place preference learning” was allowed only in the assigned corner at the restricted times. One corner was assigned for each four mice and only these mice got water in the respective corner. At completion of the conditioning, the corner in which the water-reward was applied was switched to the respective opposite corner and the re-learning of the newly assigned corner was tested for another 7 days. During this “place preference reversal” water access was confined to the 11–12 a.m. and 4–5 p.m. drinking times. At completion, the IntelliCages were cleaned and cues removed.

Finally, mice learnt to get water in one corner in a clockwise or anti-clockwise sequence of the corners (Endo et al., 2011). They were again adapted to the restricted drinking times for 4 days before starting this session because cleaning of the cages had removed the environmental cues. At the start of the chaining protocol mice could start drinking in an arbitrary corner during the restricted drinking times. The next corner, where they could drink, was then in a clockwise or anti-clockwise sequence. Each 4 mice of each group were assigned to either chaining. After learning the chaining behavior for 8 days the direction of the chaining was reversed in that clockwise trained mice got water in anti-clockwise direction and vice versa. The reversal chaining behavior was assessed for another 10 days.

### Statistics

Behavioral data are presented as mean ± s.e.m unless stated otherwise and were analyzed with SPSS 21 and Graphpad Prism. To assess the time dependent increase of the error rates independently of the inter-individual differences of the number of visits and nosepokes all events of the respective group were combined and plotted as cumulative percent errors (i.e., percent errors relative to all events) over time according to Kaplan Meier plots. For comparison, a non-parametric log-rank (Mantel-cox) test was used. In addition we used linear regression analyses for comparison of the slope of the cumulative error curves except for the avoidance acquisition which were best fitted according to non-linear saturation.

The time courses of the daily error percentages were submitted to analysis of variance for repeated measurements (rm-ANOVA and multifactorial rm-ANOVA). The within subject factor was “time,” between subject factors were “treatment” (i.e., sham vs. SNI), “age” (i.e., young vs. aged), and “genotype” (C57BL6/J vs. Grn^−/−^ mice). In addition, we used “group” as between subject factor (i.e., sham-young, SNI-young, sham-aged, and SNI-aged) separately for the genotypes. Although experiments in C57BL/6 mice and progranulin deficient mice were performed sequentially, the protocols were identical. Therefore, “genotype” was also used as between subject factor or added as a covariate. In case of significant differences of ANOVAs, groups were mutually compared with *t*-tests employing a correction of alpha according to Dunnett, in which the sham-treated young mice of the respective genotype were used as the reference group.

## Results

### Nociception

**Figure 2:**
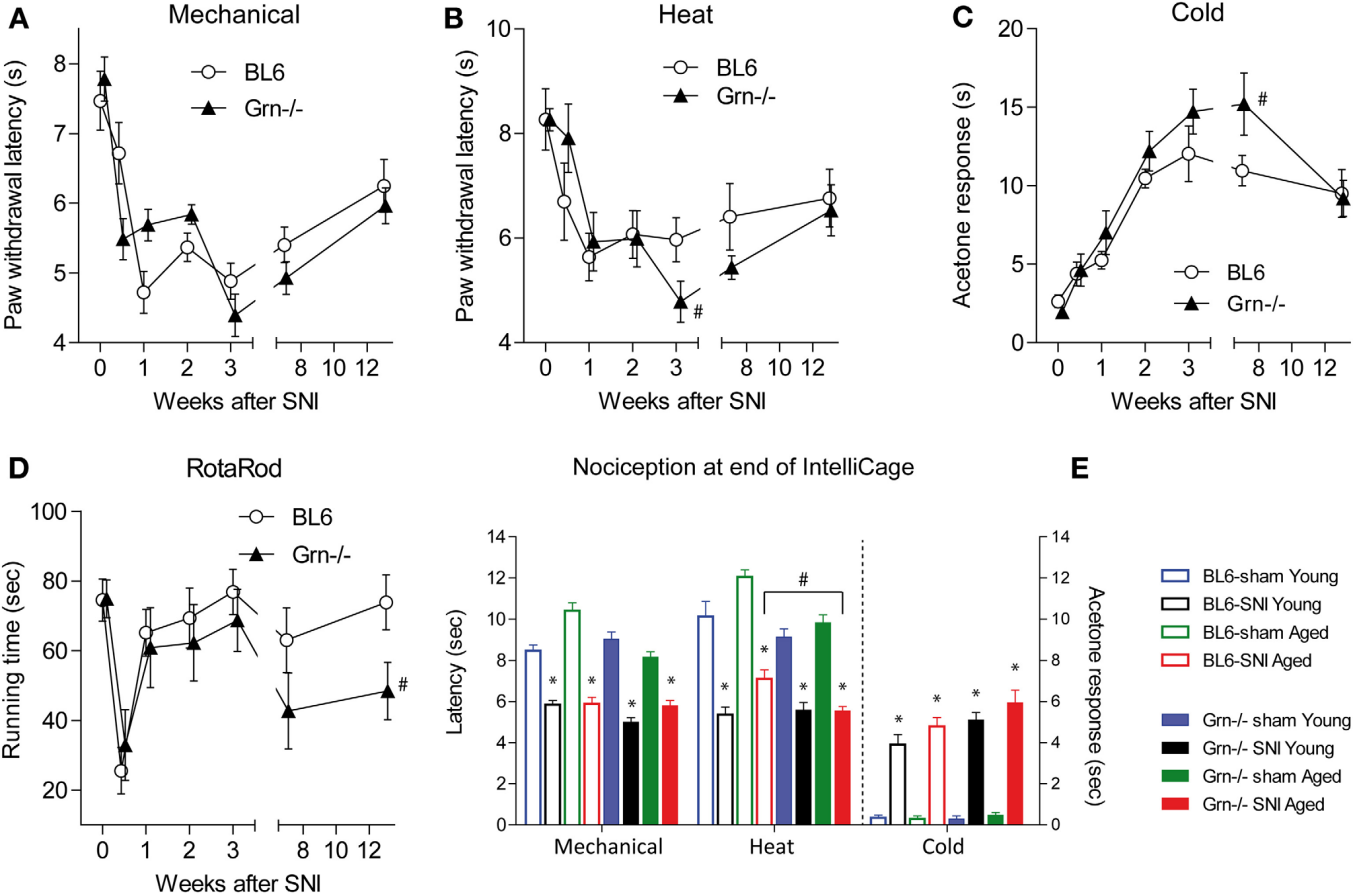
**Nociceptive behavior and motor functions in C57BL6/J and progranulin deficient (Grn^−/−^) mice before and after injury of the sciatic nerve in the Spared Nerve Injury (SNI) model. (A–C)** Time course of the nociceptive behavior upon mechanical, heat and cold stimulation before and after SNI in young C57BL6/J and progranulin deficient (Grn^−/−^) mice. **(D)** RotaRod running behavior before and after SNI. **(E)** Nociceptive behavior at the end of the IntelliCage experiments after sham or SNI surgery in young and aged C57BL6/J and progranulin deficient mice. The asterisks (^*^) denote significant differences between the respective SNI and sham treated mice, and the crosses (#) show significant differences between genotypes, *P* < 0.05. Data are means ± s.e.m. of *n* = 8–12 per group for all tests.

Nociceptive hypersensitivity after SNI developed within 1 week and was maintained for several months encompassing the period of the IntelliCage experiments (Figures 2A–C,E) (rm-ANOVA factor “time” for mechanical *F* = 23.92, *df* = 6, *P* < 0.001; for heat *F* = 8.80, *df* = 6, *P* < 0.001; for cold *F* = 27.45, *df* = 6, *P* < 0.001). In contrast, RotaRod running behavior recovered within 1 week after SNI showing that IntelliCage experiments were not confounded by substantial motor function deficits (Figure 2D). The time course of SNI-evoked nociceptive hypersensitivity was similar in C57BL6/J and progranulin deficient mice, which showed mildly heightened SNI-evoked heat and cold hypersensitivity (Figures 2B,C). IntelliCage experiments were performed within the time frame of constant nociceptive hypersensitivity. Time course experiments were performed in a separate group of animals because animals must not be disturbed during the IntelliCage experiments. However, for further confirmation of ongoing pain, nociception was assessed at completion of the IntelliCage experiments. SNI-evoked neuropathic pain was maintained in young and aged mice (Figure 2E). Paw withdrawal latencies of the paw ipsilateral to the sciatic nerve lesion were reduced upon mechanical and heat stimulation as compared to the contralateral side (not shown) and as compared to sham treated mice in all groups and acetone evoked cold pain responses were enhanced as compared to sham treated mice in all groups. Statistical results of posthoc tests are depicted in Figure 2E. Progranulin deficient aged mice showed an enhanced SNI-evoked nociceptive hypersensitivity for thermal stimuli (rm-ANOVA with “stimulus” as within subject factor and “genotype” as between subject factor: *F* = 12.59, *df* = 7, *P* < 0.001, posthoc for heat *P* < 0.05). The young SNI-treated mice did not significantly differ between genotypes at that time point.

### Place avoidance acquisition

**Figure 3:**
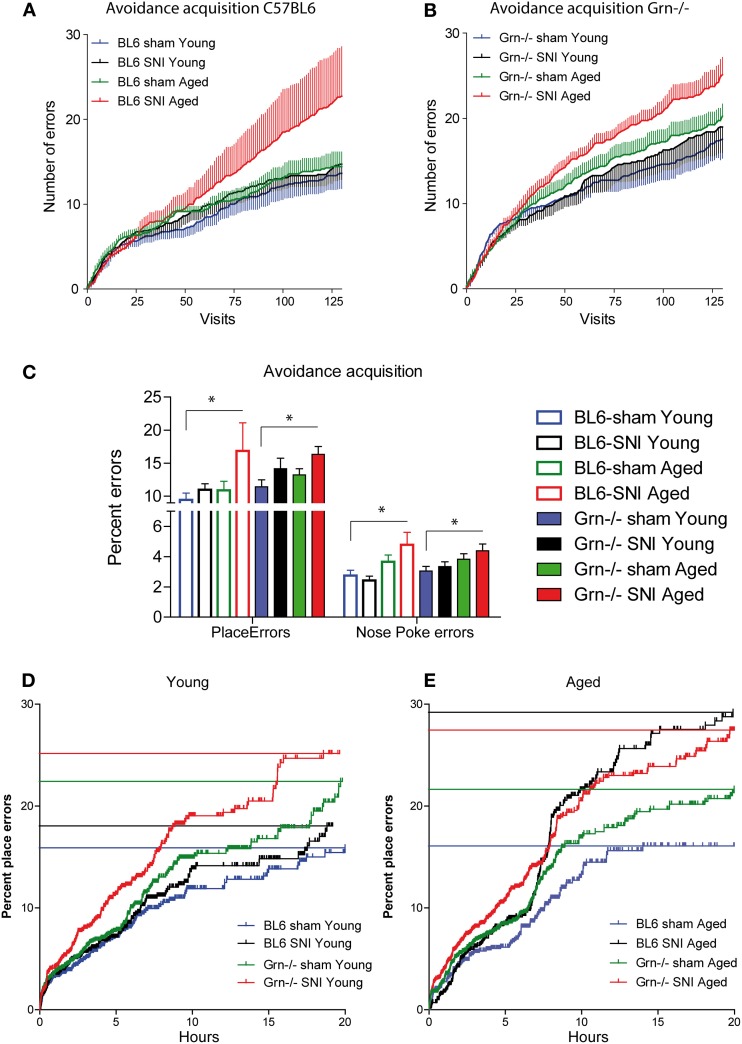
**Avoidance acquisition in the IntelliCage in young and aged C57BL6/J and progranulin deficient (Grn^−/−^) mice. (A,B)** Mean ± s.e.m cumulative place errors per visit number in sham and SNI-treated (Spared Nerve Injury of the sciatic nerve) in young and aged C57BL6/J and progranulin deficient mice (*n* = 8 per group). **(C)** Group means of error percentages during the 24 h-avoidance acquisition period for place errors and nosepoke (NP) errors. The asterisks denote significant differences vs. the reference group (sham-treated young) with *P* < 0.05, *n* = 8. **(D,E)** Cumulative percentages of place errors over time in young and aged mice of both genotypes during avoidance acquisition. All events of the group were combined and plotted according to Kaplan Meier curves. Each vertical line is an error and the horizontal lines indicate the percent error estimates of the respective groups, where 25% (one wrong corner out of four) would be the random level.

In the 24h-avoidance acquisition mice received an air-puff punishment in one corner. Most animals learned to avoid the corner, in which air-puffs were applied (Figure 3), i.e., had an error rate <25% at the end of the acquisition period, except each two in the groups of SNI-treated aged mice of both genotypes. These mice behaved normally in subsequent tasks. Progranulin-deficient mice tended to make more place errors than the control mice in all treatment groups. Mean cumulative errors per visit number are shown in Figures 3A,B. In aged mice, SNI caused a significant increase of the error rates in both genotypes (Figure 3C 24 h-means, E cumulative), with a similar result for place errors (wrong corner) and nosepoke errors.

In young mice the SNI-effect manifested only in progranulin deficient mice (Figure 3D cumulative). The 24-h means for place errors (wrong corner) and nosepoke errors, however, were similar in all young groups (Figure 3C). ANOVA revealed significant differences between groups (univariate ANOVA of 8 groups for 24 h mean error rates; *F* = 2.630; *df* = 7, *P* = 0.020). Results of posthoc analyses are shown in Figure 3C. Some animals stopped nose-poking completely after receiving punishments and the analysis of avoidance acquisition was therefore focused on place errors.

### Extinction of place avoidance

**Figure 4:**
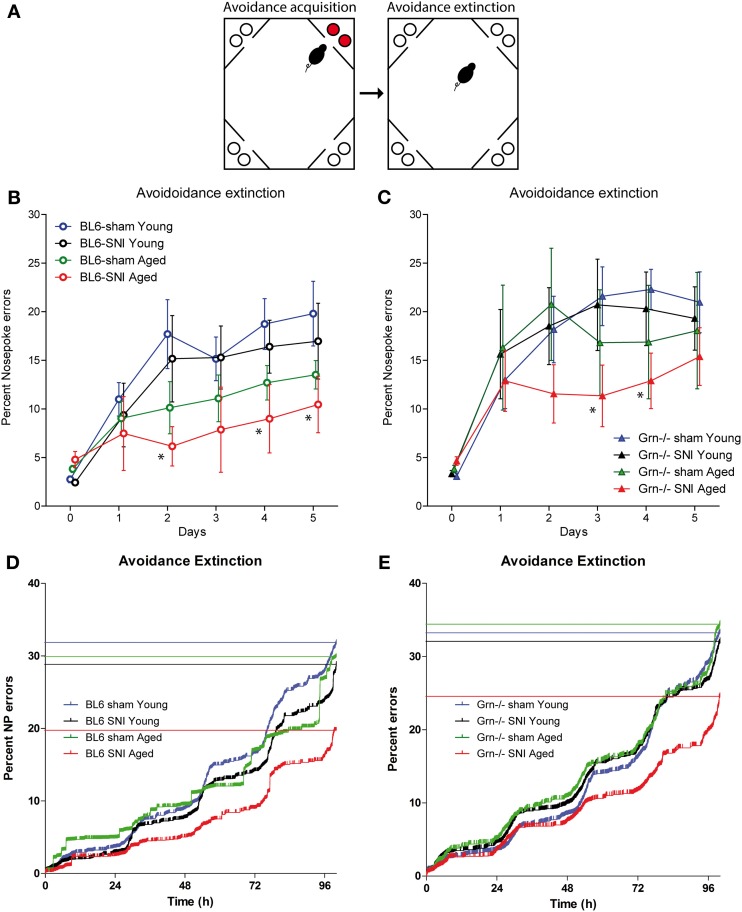
**Avoidance extinction in the IntelliCage in young and aged C57BL6/J and progranulin deficient (Grn^−/−^) mice. (A)** Illustration of the avoidance acquisition and extinction tasks. Red circles indicate nosepokes where air-puff punishments were applied and water access was blocked. White circles show free water access. **(B,C)** Time courses of nosepoke error percentages (mean ± s.e.m) per day in C57BL6/J **(B)** and progranulin deficient (Grn^−/−^) mice **(C)**; *n* = 8 per group. Time “0” shows the error proportion achieved during the 24 h acquisition period (mean over 24 h). rm-ANOVAs revealed significant differences between groups. SNI-treated aged mice of both genotypes showed the lowest error rates, *P* < 0.05. Asterisks denote time points which differed significantly vs. sham treated young mice, which were used as the reference group (*P* < 0.05, Dunnett *post hoc*). **(D,E)** Cumulative nosepoke error rates over time in young and aged mice during avoidance extinction. The step-wise day-to-day increase is due to preferred drinking times in the morning. All events of the group were combined and plotted according to Kaplan Meier curves. Each vertical line is an error and the horizontal lines indicate the percent error estimates of the respective groups. Aged mice of both genotypes showed the lowest nosepoke error rates i.e., the strongest retention of the aversive memory.

The time courses of the extinction of the aversive memory were tested by re-allowing water access in each corner without air-puff punishments (task illustration Figure 4A). Acquisition and extinction phases were separated by a 24 h home cage period with water deprivation in the last 18 h to ensure immediate search for water despite the previous punishments. All animals showed an increase of the place and nosepoke error rates as compared to the mean error rate of the acquisition period (Figures 4B,C) (rm-ANOVA within subject factor “time”: *F* = 42.272, *df* = 5, *P* < 0.001). The increase of the nosepoke error rate was stronger in young than aged mice (“time × age”: *F* = 4.670, *df* = 5, *P* < 0.001) and the memory for the aversive place was most strongly retained in SNI-treated aged mice (Figure 4) (“time × groups” i.e., sham-young, SNI-young, sham-aged, SNI-aged: *F* = 2.136, *df* = 15, *P* = 0.009). The latter showed the lowest cumulative nosepoke error rates (Figures 4D,E) indicating that those mice, which had shown the worst acquisition of aversive memory, showed the strongest retention thereof. This preservation of aversive memory was observed in both genotypes (interaction: “age × treatment × genotype” *F* = 0.149, *df* = 1, *P* = 0.700). Analysis across treatment groups and ages revealed higher error rates in progranulin deficient mice as compared to the controls (rm-ANOVA for “genotype” *F* = 4.026; *df* = 1; *P* = 0.0492).

### Conditioned place preference learning

**Figure 5:**
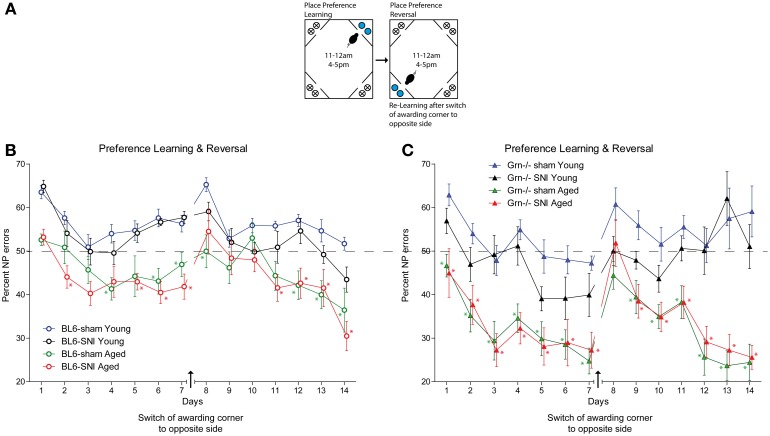
**Place “preference learning” and “reversed place preference learning” in young and aged C57BL6/J and progranulin deficient (Grn^−/−^) mice. (A)** Illustration of the place preference learning and reversal tasks. The blue circles indicate nosepokes where a water award was provided. The crossed circles show non-awarding nosepokes, i.e., water access was blocked, without punishment. **(B,C)** Time courses of nosepoke error percentages (mean ± s.e.m) per day in C57BL6/J and progranulin deficient (Grn^−/−^) mice, *n* = 8 per group, during place preference learning and reversed learning. rm-ANOVA revealed significant differences between young and aged mice in both genotypes, *P* < 0.05, *n* = 8 per group. Time points which differed significantly vs. the sham treated young mice are indicated per asterisks, *P* < 0.05.

During this session mice learnt to get water in a specified corner after performing a nosepoke during restricted “drinking times” (task illustration Figure 5A). The time restrictions provided a synchronization of the drinking behavior. After conditioning to one corner, the awarding corner was switched to the opposite corner. All treatment groups were able to perform this task and had error rates below the 75% random level (Figures 5B,C). There was an improvement over time in all groups both in the first phase and in the reversal phase (rm-ANOVA for “time” for both phases *P* < 0.001), but the aged animals of both genotypes showed lower error rates than the young mice (Figures 5B,C) (rm-ANOVA factor “age” *F* = 63.758, *df* = 1, *p* < 0.001). SNI did not affect the place preference learning behavior, neither in young nor aged mice and neither in control nor progranulin-deficient mice (rm-ANOVA “treatment”: *F* = 1.785, *df* = 1, *P* = 0.178). Aged progranulin deficient mice showed stronger preference of the awarded corner than the respective aged controls, irrespective of SNI treatment (rm-ANOVA for the interaction “time × age × genotype” *F* = 3.146, *df* = 44, *P* = 0.002).

Upon switching the awarding corner to the opposite site (“place preference reversal”), the error rates temporarily increased. The time needed to recover preference was similar in all groups except for young progranulin deficient mice, in which error rates remained high up to the end of the reversal phase (Figure 5C). Aged mice of both genotypes recovered stronger preference of the awarded place than young mice (rm-ANOVA factor “age” *F* = 51.668, *df* = 1, *P* < 0.001), which was, however, stronger in progranulin deficient aged mice (rm-ANOVA for the interaction “age × genotype” *F* = 5.992, *df* = 1, *P* = 0.018). Nerve injury had no significant impact on the “reversal preference learning” in neither genotype. Overall, variability was higher in progranulin deficient mice than in controls. Figure 5 indicates the time points at which error rates were significantly different from those of young sham-treated mice, which were used as the reference group (comparison of 4 groups separately for each genotype)*.*

### Chaining and reversed chaining

**Figure 6:**
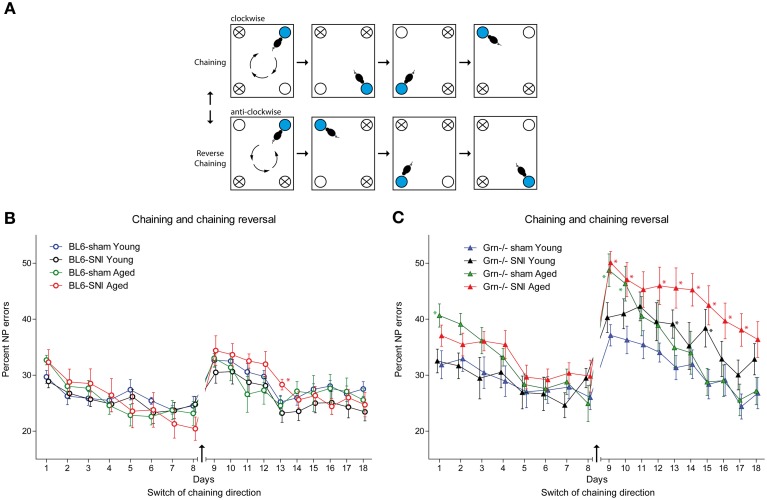
**Spatial sequence learning in young and aged C57BL6/J and progranulin deficient (Grn^−/−^) mice. (A)** Illustration of the “chaining” and “reversal of chaining” tasks. Mice were randomly allocated to either starting with the clockwise or anti-clockwise direction. After 8 days the direction was conversed. The blue circle indicates the awarding corner and the white circle is the corner, in which the next water award was applied. The crossed circles show the corners where water access was denied. **(B,C)** Time courses of nosepoke error percentages (mean ± s.e.m) per day in C57BL6/J and progranulin deficient (Grn^−/−^) mice, *n* = 8 per group during “chaining” and “reversal of chaining.” rm-ANOVA revealed significant differences between SNI-treated aged mice as compared to young mice, *P* < 0.05. In progranulin deficient mice, SNI also caused an increase in the error rate in young mice. Time points which differed significantly vs. the sham treated young mice are indicated per asterisks, *P* < 0.05.

In this task mice had to learn a clockwise or anti-clockwise sequence of awarded corners, the direction of which was switched after 8 days (Figure 6A task illustration). Drinking was only allowed in the specified periods (11–12 a.m. and 4–5 p.m.). Progranulin deficient mice had higher nosepoke error rates in all treatment groups as compared to the controls, which became most obvious after reversal of the chaining direction (Figure 6C) and was stronger in aged than young mice. rm-ANOVA revealed significant differences between genotypes (*F* = 26.108, *df* = 1, *P* < 0.001). In C57BL6/J mice all treatment groups were similar during chaining and reversal thereof (Figure 6B), but SNI-treated aged mice tended to show the worst performance after reversal of the chaining direction. The effect of SNI was stronger in progranulin deficient mice and also affected young SNI-treated progranulin deficient mice (Figure 6C) (rm-ANOVA for “time × age × treatment × genotype” *F* = 1.625, *df* = 17, *P* = 0.05). While all C57BL6/J mice were able to learn the reversed direction within 4 days and returned to pre-reversal error rates, progranulin deficient SNI-treated mice failed to recover this error rate. Overall, aged mice had higher error rates than young mice (rm-ANOVA for “age” *F* = 3.564, *df* = 1, *P* = 0.067) and SNI-treated mice tended to make more errors than sham treated mice (rm-ANOVA for “treatment” *F* = 3.38, *df* = 1, *P* = 0.072, “age × treatment” n.s.). Figure 6 indicates the time points at which error rates for treatment groups were significantly different from those of young sham-treated mice, which were used as the reference group (comparison of 4 groups separately for genotypes).

In summary, the behavioral IntelliCage experiments revealed that SNI in aged mice blocked the extinction of aversive memory, and that progranulin deficiency in combination with SNI seriously impaired the learning of spatial sequences.

### Visits and lickings

The number of daily visits and nosepokes did not show differences between SNI and sham treated mice in neither genotype nor age (not shown). However, in C57BL6/J mice, young mice visited corners more frequently than aged C57BL6/J mice (rm-ANOVA Visits: *F* = 15.1, *df* = 3, *P* < 0.001). Daily visits were, however, similar in young and aged progranulin deficient mice.

## Discussion

In the present study we assessed the impact of chronic neuropathic pain on learning and memory in mice and the influence of age based on the hypothesis that chronic pain may narrow cognition and experience, which in turn, may further increase pain. We used progranulin deficient mice as a pro-aging model, because they develop premature signs of brain aging (Ahmed et al., 2010; Yin et al., 2010a,b). Our hypothesis was supported by recent studies, which found a decline of cognitive functions in rats with neuropathy (Gregoire et al., 2012; Cardoso-Cruz et al., 2013) and in patients with persistent pancreatic pain (Jongsma et al., 2011).

After SNI, all mice had persistent and relative constant nociceptive hypersensitivity throughout the experiments. Our results show that young mice with sciatic nerve injury behave normally in all tasks i.e., learning and memory was not impaired by chronic pain in young mice. However, aged mice with SNI needed longer to learn avoidance of one punishing corner. However, once learnt, the aged SNI-treated mice of both genotypes showed the slowest extinction of aversive memory suggesting that ongoing pain in these aged mice may deepen the memory for unpleasant experiences. The result agrees with previous studies in rodents, which demonstrated a long-term maintenance of aversive memory in rats with chronic pain (Hummel et al., 2008) and inability of mice with SNI to extinguish contextual fear (Mutso et al., 2012). If translated into the human situation this could mean that chronic pain may strengthen the memory for unpleasant experiences, possibly because pain may absorb attention and narrow cognition so that learning of novel information may be compromised (Lotsch et al., 2012). Such adaptations likely involve multiple signaling networks, particularly of the cingulate cortex, anterior insula cortex and amygdala. In addition, specific dopaminergic neurons in the ventral tegmental area are involved in aversive and appetitive learning (Cohen et al., 2012; Lammel et al., 2012) and are important sites of opioid mediated analgesia and reward (Ewan and Martin, 2011).

In contrast to aversive learning, nerve injury did not negatively impact on reward-mediated place preference learning in neither genotype nor age group. Indeed, aged mice with or without nerve injury showed a stronger preference of the awarded corner than the young mice, which agrees with a previous study in the IntelliCage (Mechan et al., 2009). The age pattern was maintained throughout conditioning to one corner and reversal to the opposite corner and was obvious in both genotypes. Old age may increase the subjective cost of errors i.e., extra visits and locomotion, thereby increasing the motivation to learn. Old age may also negatively impact on exploratory drive and thereby seemingly reduce errors. This was also suggested by comparison of daily visits, which were higher in young than aged C57BL6/J mice. This age pattern of daily visits, however, was not evident in progranulin deficient mice. Alternatively old age might also increase the appetitive drive of the water award.

Award was also the driving force in the chaining experiment. In this spatial sequencing task the aged progranulin deficient mice with nerve injury, but not those with a sham injury, had very high error rates after switching the direction of clockwise-anticlockwise chaining. The effect of nerve injury was also evident in young progranulin deficient mice but was not obvious in the C57BL6/J mice, suggesting that this was an effect of “genotype + nerve injury.” The observation suggested that nerve injury in combination with progranulin deficiency may reduce the cognitive flexibility and ability to adjust behavior to novel situations involving right-left discrimination. In contrast to place preference learning, which is cognitively not demanding and learnt without problems even by mice with hippocampal lesions (Voikar et al., 2010), chaining reversal is the cognitively most challenging of the tests so that is not surprising that deficits emerged in this test. The deficits were most obvious in aged progranulin deficient SNI-treated mice. It may be hypothesized that the sciatic nerve injury on one side and the subsequent restructuring of sensory and motor neuronal networks might be particularly unfavorable for tasks requiring discrimination of right and left. Progranulin deficient mice likely lose more neurons after axonal injury because lack of this neurotrophic factor reduces the ability of the neurons to survive the neuronal stress (Van Damme et al., 2008; Gao et al., 2010; Gass et al., 2012; Lim et al., 2012). The importance of progranulin may increase with age. An enhanced neuronal loss may cause more profound alterations of synaptic connectivity, which may add on to the subtle alterations of synapse function, which are present in progranulin deficient mice at baseline (Tapia et al., 2011; Petkau et al., 2012). This may also contribute to the mildly enhanced thermal nociceptive hypersensitivity in progranulin deficient mice and explain the observed progranulin-dependent difficulties in right-left discrimination after SNI.

In summary, aged mice with chronic neuropathic pain showed the strongest maintenance of aversive memory as compared to sham treated mice and as compared to younger SNI-treated mice suggesting that chronic pain at old age may heighten the susceptibility for unpleasant experiences and possibly vice versa. In addition, progranulin deficient mice with nerve injury showed impaired right-left discrimination learning. The unfavorable alliance of “age and nerve injury” and of “progranulin deficiency and nerve injury” suggests that nerve injury evoked chronic pain in combination with old age may impinge on the ability to give up accustomed behavior and adapt it to novel situations.

### Conflict of interest statement

The authors declare that the research was conducted in the absence of any commercial or financial relationships that could be construed as a potential conflict of interest.
